# Supplementary figures and images for: A Multidisciplinary Approach Reveals an Age-Dependent Expression of a Novel Bioactive Peptide, Already Involved in Neurodegeneration, in the Postnatal Rat Forebrain
Source: Brain Sci. 2018 Jul 10;8(7):132. doi: 10.3390/brainsci8070132 (PMC6070872; doi:10.3390/brainsci8070132)

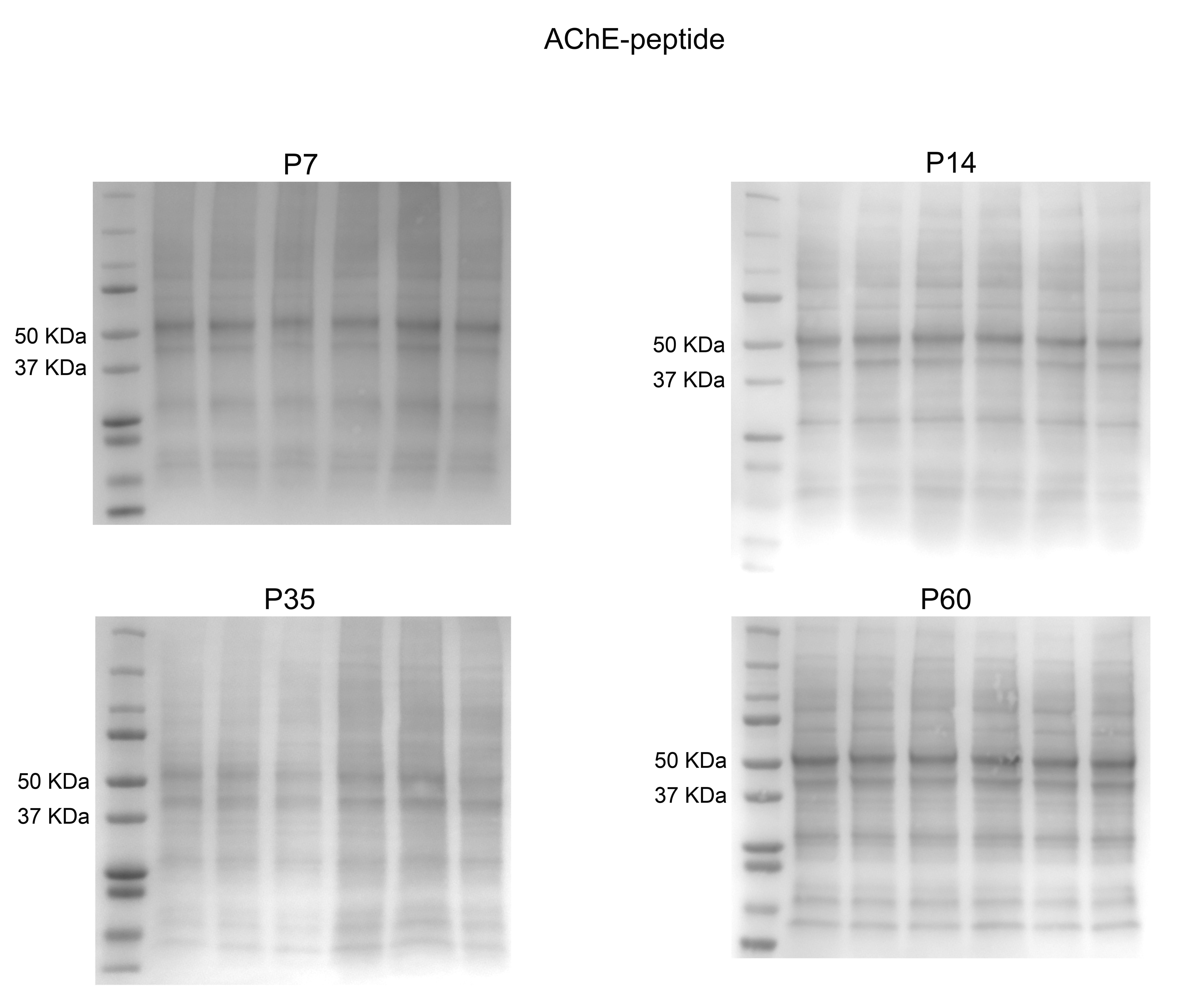

Supplement: Supplementary file 1 [file brainsci-08-00132-s001.zip › Supplementary figure 1.jpg]

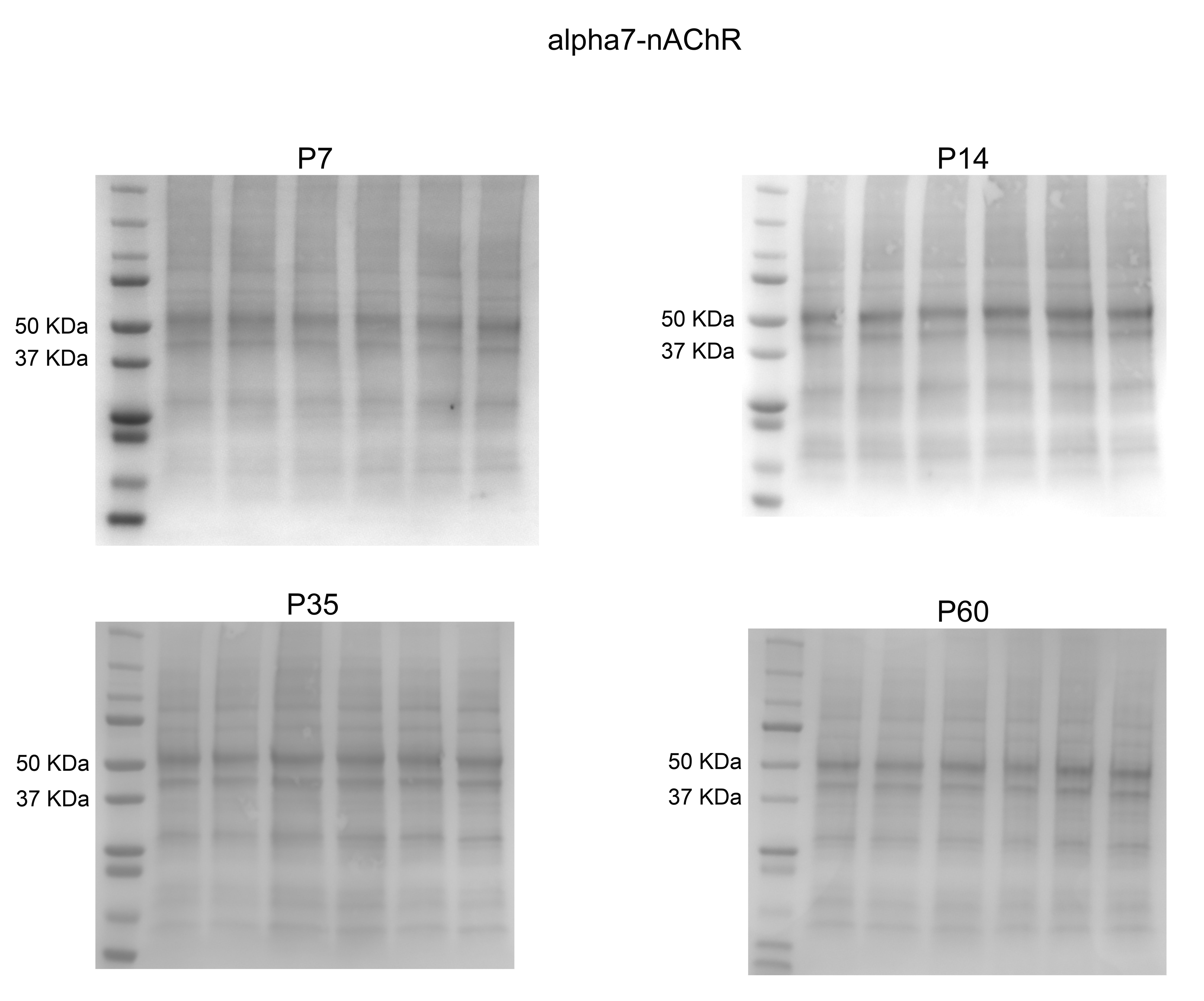

Supplement: Supplementary file 1 [file brainsci-08-00132-s001.zip › Supplementary figure 2.jpg]

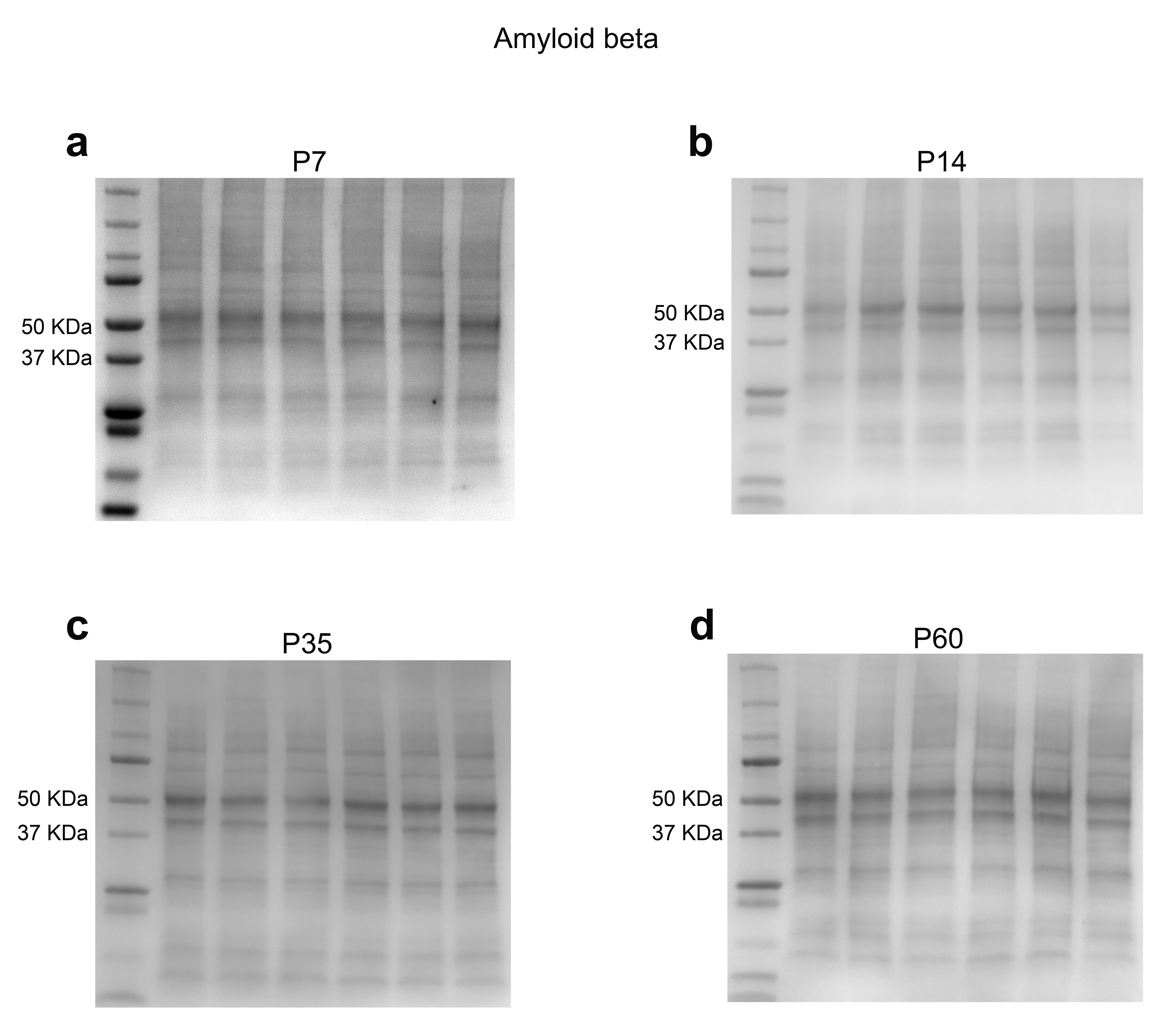

Supplement: Supplementary file 1 [file brainsci-08-00132-s001.zip › Supplementary figure 3.jpg]

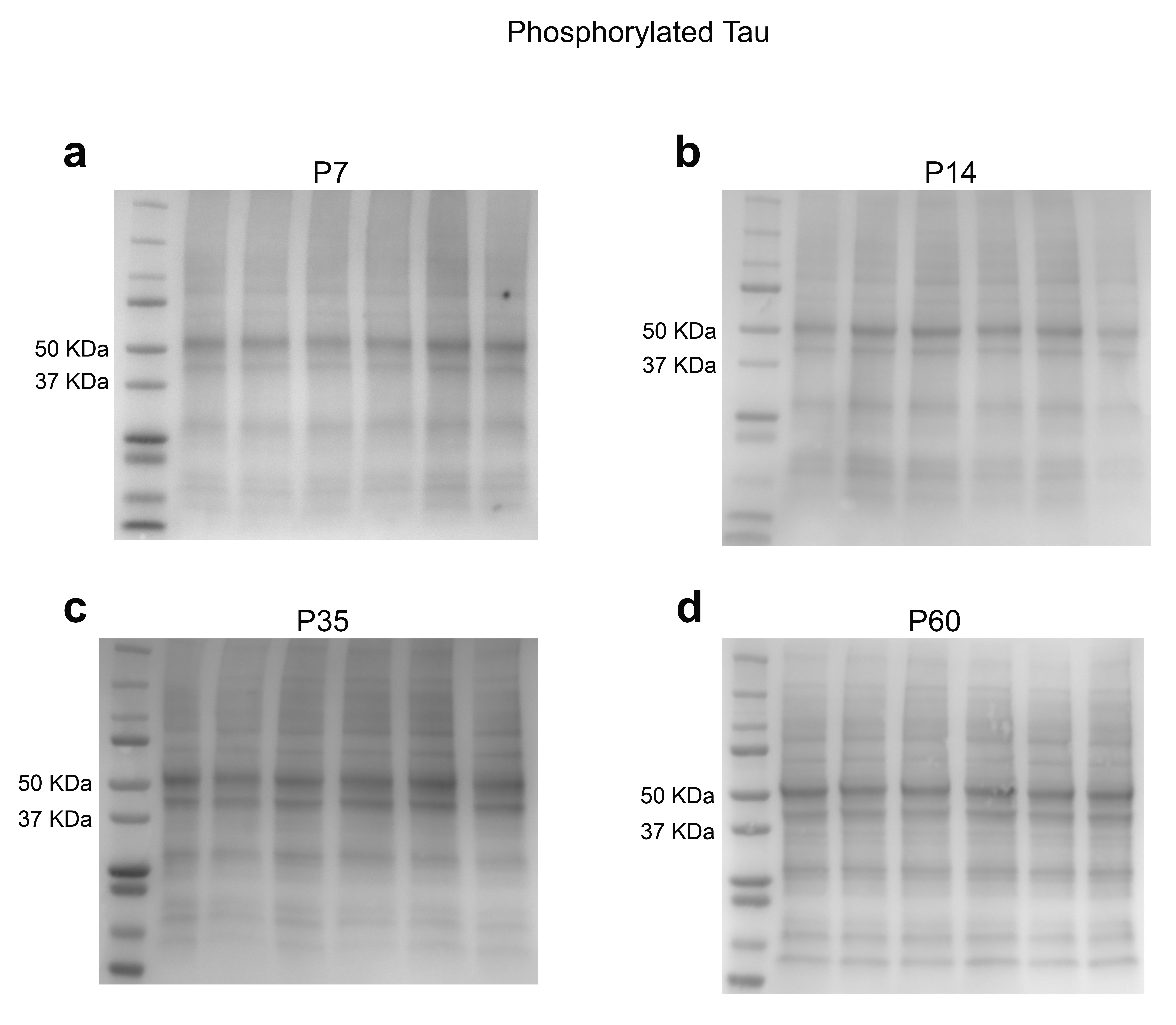

Supplement: Supplementary file 1 [file brainsci-08-00132-s001.zip › Supplementary figure 4.jpg]

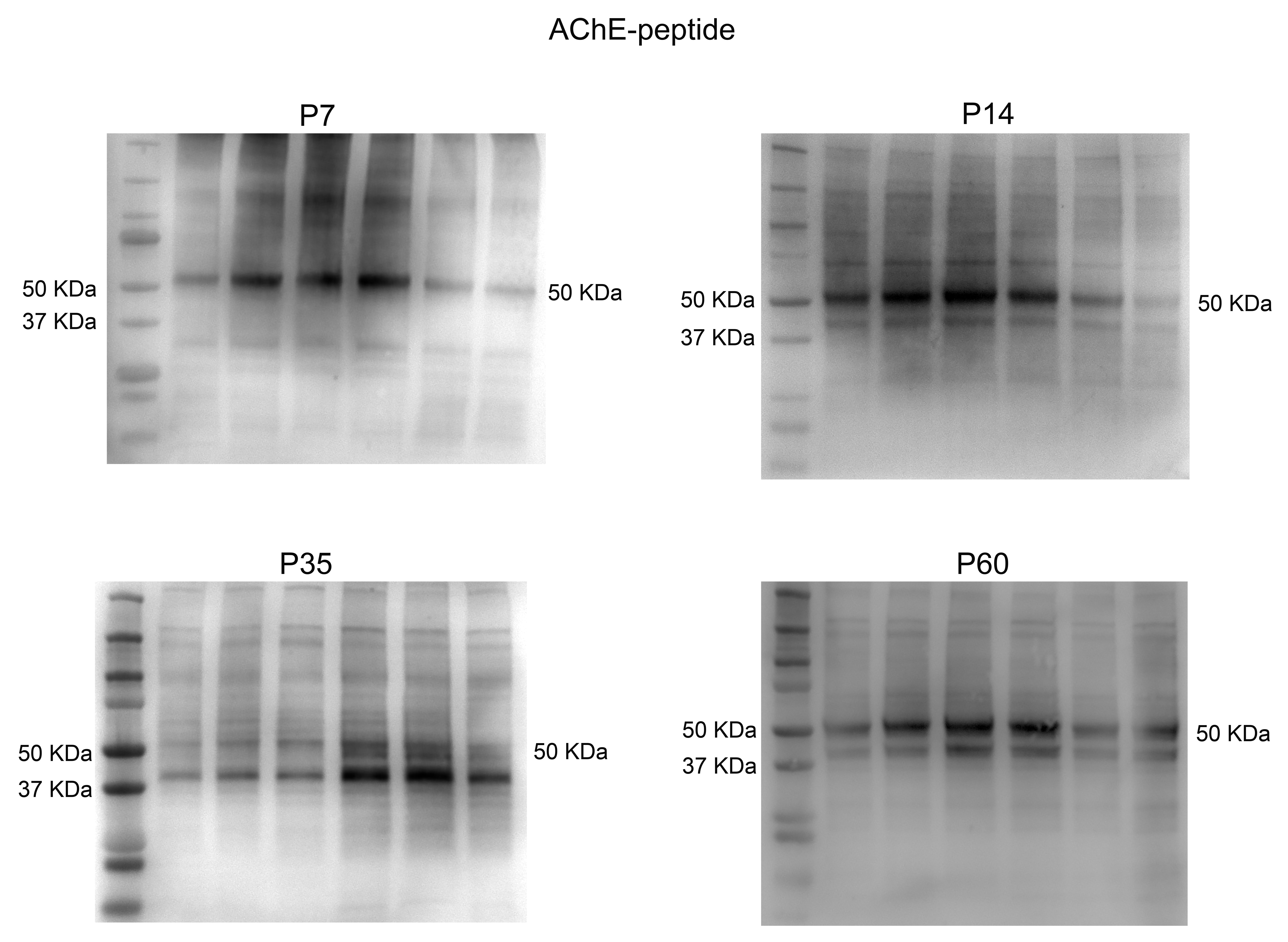

Supplement: Supplementary file 1 [file brainsci-08-00132-s001.zip › Supplementary figure 5.jpg]

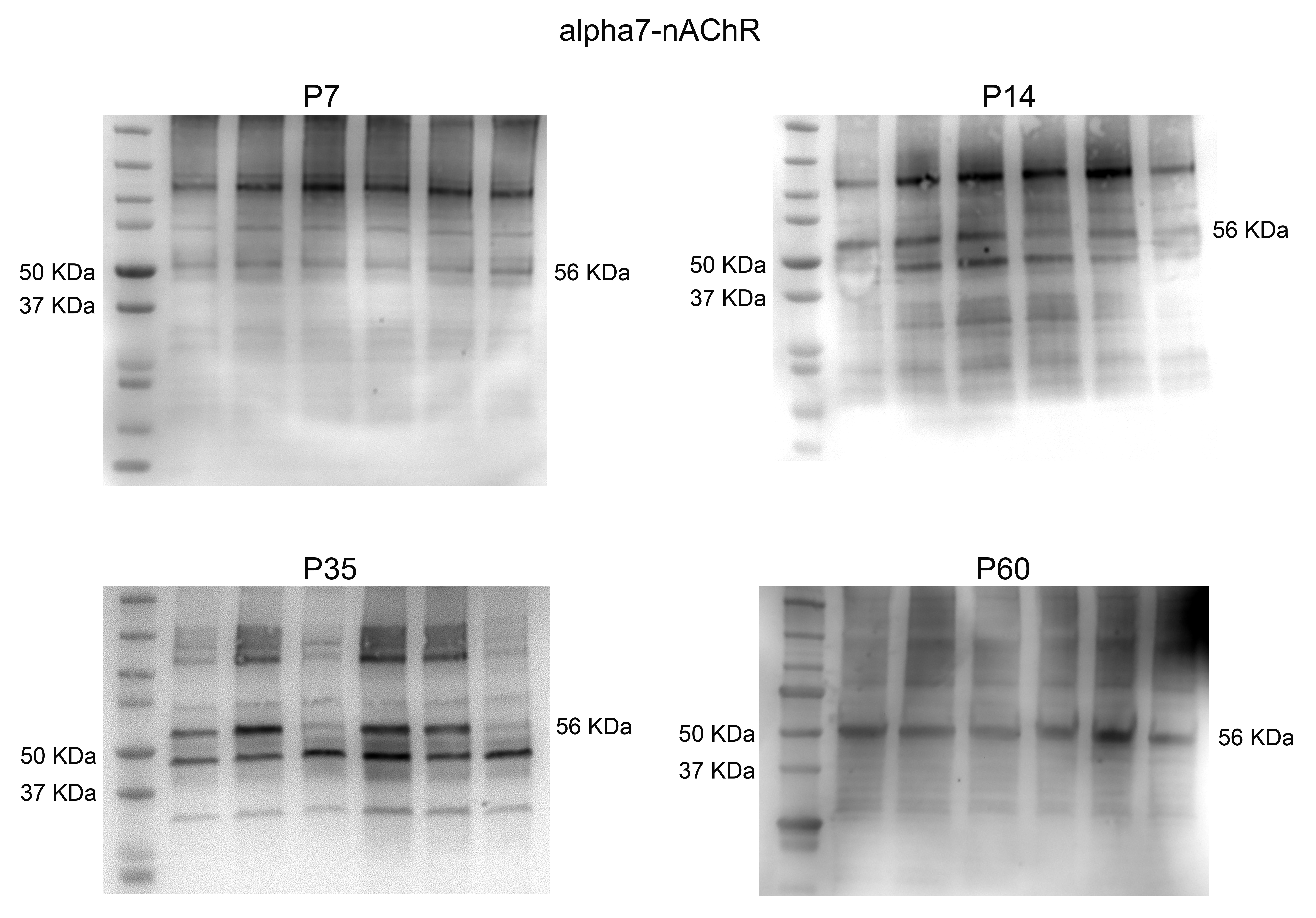

Supplement: Supplementary file 1 [file brainsci-08-00132-s001.zip › Supplementary figure 6.jpg]

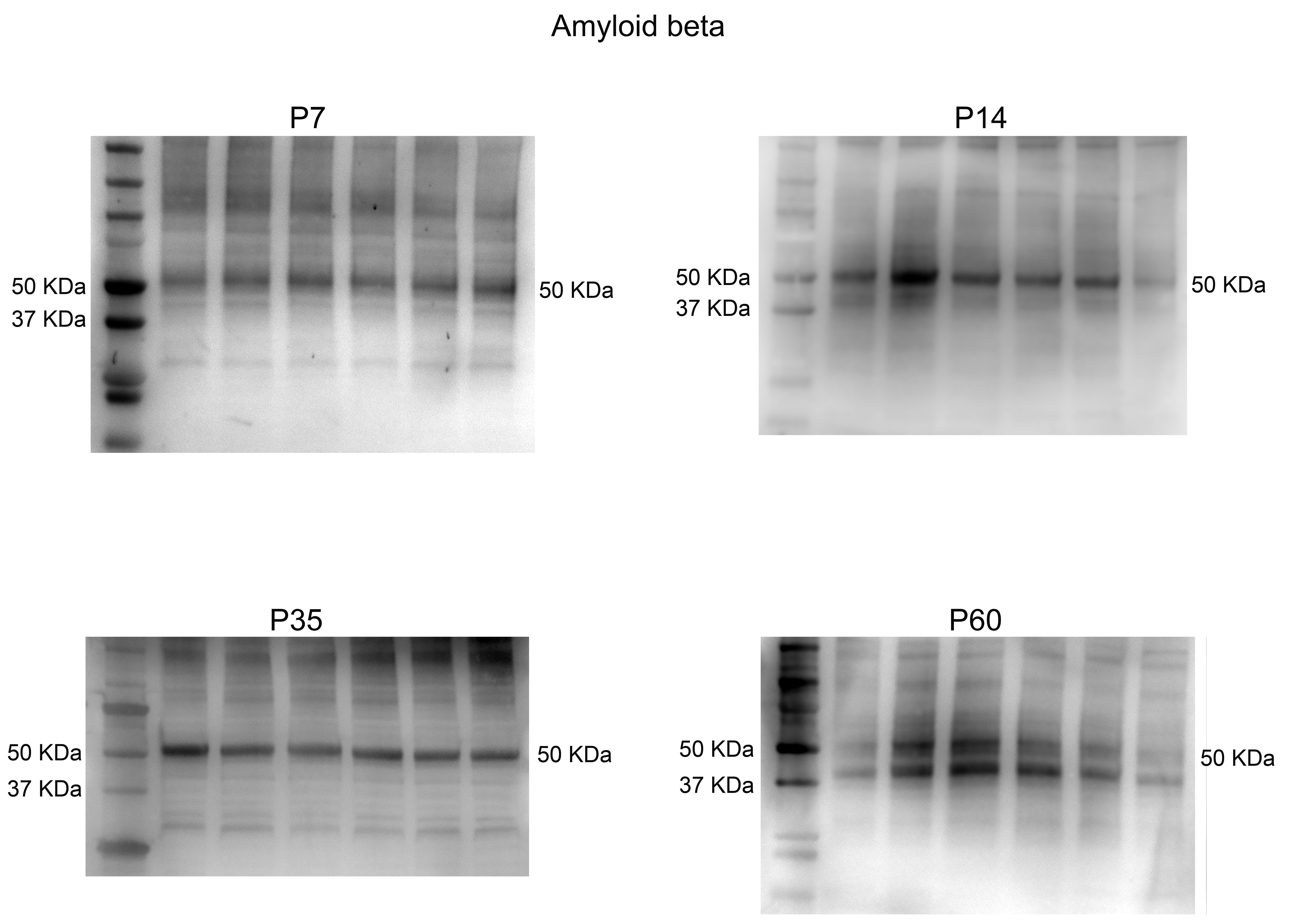

Supplement: Supplementary file 1 [file brainsci-08-00132-s001.zip › Supplementary figure 7.jpg]

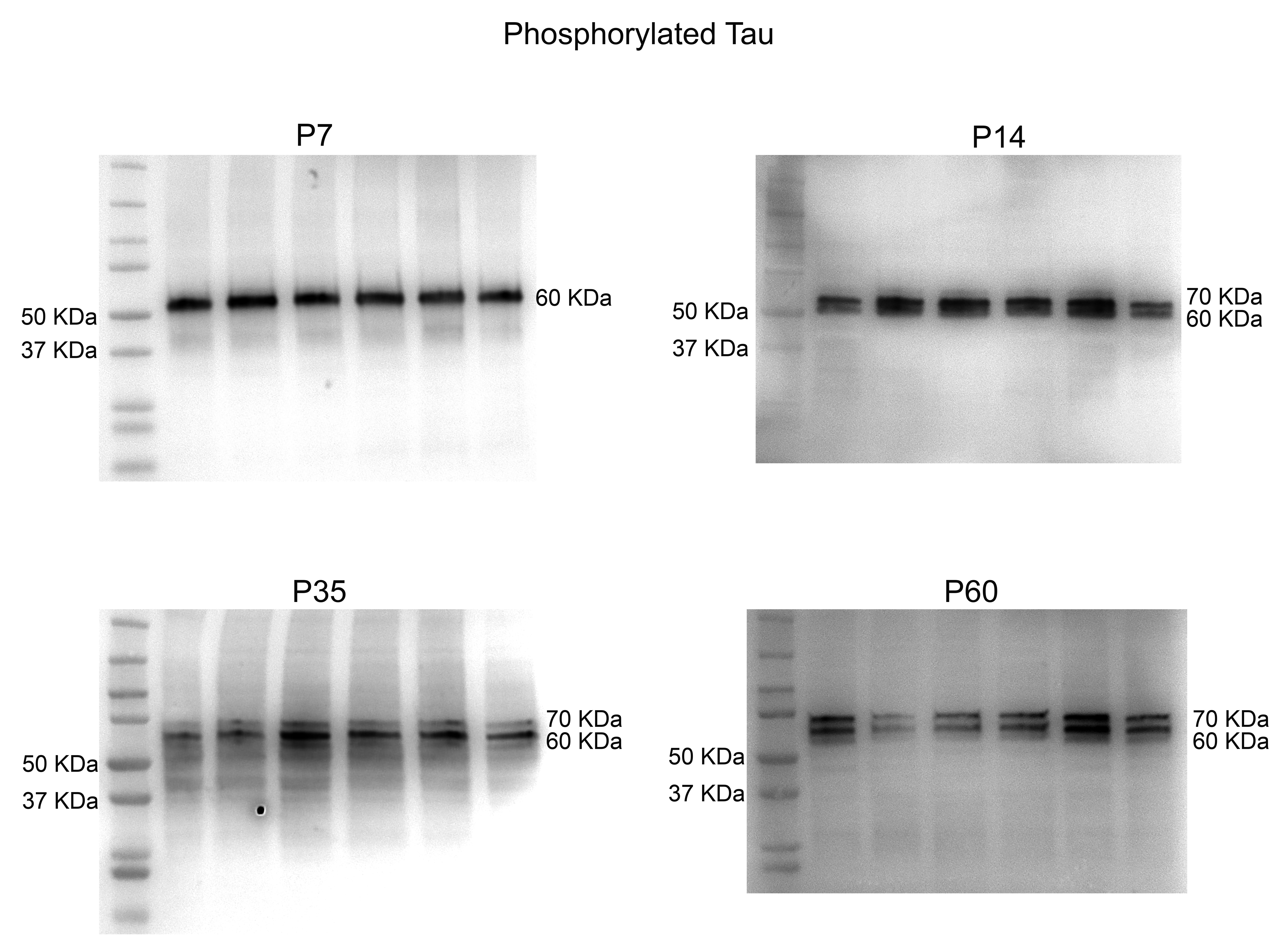

Supplement: Supplementary file 1 [file brainsci-08-00132-s001.zip › Supplementary figure 8.jpg]
